# Plastoglobules: a new address for targeting recombinant proteins in the chloroplast

**DOI:** 10.1186/1472-6750-7-4

**Published:** 2007-01-10

**Authors:** Pierre-Alexandre Vidi, Felix Kessler, Claire Bréhélin

**Affiliations:** 1 Institute of Botany, University of Neuchâtel, rue Emile Argand 11, CP158, CH-2009 Neuchâtel, Switzerland; 2 Department of Medicinal Chemistry and Molecular Pharmacology, Purdue University, 575 Stadium Mall Dr., RHPH 210, West Lafayette, IN 47907, USA

## Abstract

**Background:**

The potential of transgenic plants for cost-effective production of pharmaceutical molecules is now becoming apparent. Plants have the advantage over established fermentation systems (bacterial, yeast or animal cell cultures) to circumvent the risk of pathogen contamination, to be amenable to large scaling up and to necessitate only established farming procedures. Chloroplasts have proven a useful cellular compartment for protein accumulation owing to their large size and number, as well as the possibility for organellar transformation. They therefore represent the targeting destination of choice for recombinant proteins in leaf crops such as tobacco. Extraction and purification of recombinant proteins from leaf material contribute to a large extent to the production costs. Developing new strategies facilitating these processes is therefore necessary.

**Results:**

Here, we evaluated plastoglobule lipoprotein particles as a new subchloroplastic destination for recombinant proteins. The yellow fluorescent protein as a trackable cargo was targeted to plastoglobules when fused to plastoglobulin 34 (PGL34) as the carrier. Similar to adipocyte differentiation related protein (ADRP) in animal cells, most of the protein sequence of PGL34 was necessary for targeting to lipid bodies. The recombinant protein was efficiently enriched in plastoglobules isolated by simple flotation centrifugation. The viability of plants overproducing the recombinant protein was not affected, indicating that plastoglobule targeting did not significantly impair photosynthesis or sugar metabolism.

**Conclusion:**

Our data identify plastoglobules as a new targeting destination for recombinant protein in leaf crops. The wide-spread presence of plastoglobules and plastoglobulins in crop species promises applications comparable to those of transgenic oilbody-oleosin technology in molecular farming.

## Background

Recombinant proteins are massively used in medicine as diagnostic reagents, drugs or vaccines. Moreover, the rapid discovery of new pharmaceutical proteins leads to an increased demand for their production [1]. To date, production of recombinant proteins mainly relies on microbial fermentation or on insect and mammalian cell cultures. These systems allow highly controlled manufacturing procedures essential for product quality. However, they have disadvantages in term of cost and scalability. Pathogen contamination of animal cell cultures also represent an important safety issue.

Plants are now being recognised as an alternative system for the production of recombinant proteins. Plant expression systems allow large scale production of recombinant proteins with accurate folding and assembly of protein complexes [2-5]. Importantly, plant systems offer the possibility of lowering production costs by a factor of 10 to 100 compared to traditional systems [1,6]. Chloroplasts have proven a useful cellular compartment for protein accumulation owing to their large size and number. Moreover, transplastomic plants (produced by introducing DNA into the chloroplast genome) enable high yields in recombinant proteins due to a high transgene copy number and limited epigenetic phenomena [7,8]. Contamination of wild and crop species by pollen flow is also largely circumvented by organellar transformation [7].

Important issues for industrial production of plant-derived recombinant proteins are extraction and purification [9]. Standard protocols include homogenisation of plant biomass followed by chromatographic methods. However, high abundance of secondary compounds, especially in tobacco, is problematic for chromatographic procedures. Therefore, developing a cost-effective preliminary (or alternative) purification step is required [6].

**Figure 1:**
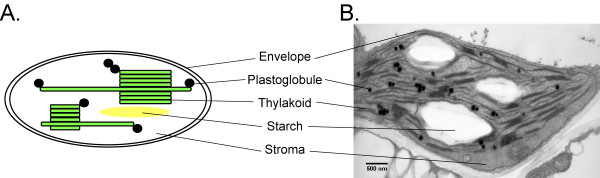
**Plastoglobule lipoprotein particles in chloroplasts**. A. Schematic diagram of a chloroplast. The organelle is delimited by a double membrane system (envelope). The interior of chloroplasts comprises the aqueous stroma, the thylakoid membranes (green) and starch granules (yellow). Plastoglobules (black circles) are lipid particles associated with thylakoids. B. Transmission electron micrograph of an Arabidopsis chloroplast. Plastoglobules are visible as dark round bodies after postfixation with osmium tetroxide.

The chloroplast has several interior compartments: the envelope membranes, the thylakoid membranes and lumen, the stroma and plastoglobules. So far, the stroma has been the major target for protein accumulation in plastids [8]. Plastoglobules are low density lipoprotein particles attached to the thylakoid membranes [10] (see Fig. 1) and coated with proteins of the PAP-fibrillin family [11-13]. The recently determined proteome [14,15] of Arabidopsis plastoglobules identified eight PAP-fibrillin proteins ("plastoglobulins").

In this study, we examined the sequence requirement for correct targeting of the plastoglobulin of 34 kDa (PGL34, [AGI: At3g58010]). We then used the genomic sequence of PGL34 to target a fluorescent protein (YFP) to plastoglobules and took advantage of the low density of plastoglobules for rapid enrichment of the recombinant protein by flotation centrifugation, YFP allowing detection throughout the procedure.

## Results

### Sequence requirement for targeting of PGL34-GFP to plastoglobules

**Figure 2:**
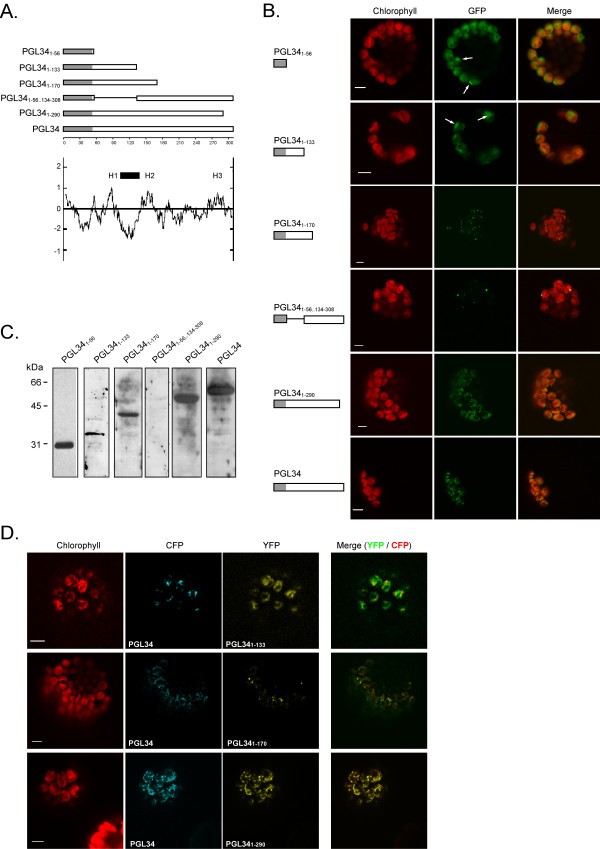
**Transient expression of truncated PGL34-GFP fusions in protoplasts**. A. DNA constructs encoding fragments of or full length *AtPGL34 *coding sequence fused to *GFP*. The transit peptide of PGL34 is shaded. The Kyte and Doolittle hydropathy plot of PGL34 is shown and domains with higher hydropathy scores (H1–H3), as well as a domain conserved among PAP-fibrillin proteins (black bar), are indicated. B. Fluorescence of the GFP fusion proteins (GFP) was detected in transformed protoplasts by confocal laser scanning microscopy. Arrows indicate strong GFP signals overlapping with weak chlorophyll autofluorescence signals (chlorophyll). Merge: overlap of chlorophyll and GFP signals. Scale bars: 5 *μ*m. C. Detection of GFP fusion proteins in transformed protoplasts by immunoblotting using anti-GFP antibodies. D. Cotransformation of protoplasts with full length and truncated PGL34. Protoplasts coexpressing *PGL34-CFP *and *PGL34*_1–133_-*YFP*, *PGL34*_1–170_-*YFP *or *PGL34*_1–290_-*YFP *were analysed by confocal microscopy. CFP fluorescence (CFP) and YFP fluorescence (YFP) were monitored sequentially using distinct excitation wavelengths and detection windows. Chlorophyll: chlorophyll autofluorescence, merge: superposition of YFP and CFP signals (green and red pseudocolours, respectively). Bar length: 5 *μ*m.

In the absence of data on the sequence requirement for plastoglobule targeting, we designed a series of C-terminal GFP fusion constructs that removed portions of *PGL34 *coding sequence (Fig. 2A). PGL34 is a representative member of the Arabidopsis PAP-fibrillin family.

Three domains with somewhat higher hydrophobic scores can be recognised in the Kyte and Doolittle hydropathy plot of PGL34 ([16], Fig. 2A). These domains comprise residues 80–94 (HI), 142–161(H2) and 273–282 (H3). Furthermore, any domain responsible for targeting or anchoring plastoglobulins to plastoglobules may be conserved among the different members of the PAP-fibrillin family. Sequence alignments (not shown) revealed that a central domain in PGL34 (residues 103 to 132, Fig. 2A) is conserved among the PAP-fibrillin family.

Both homology and hydropathic criteria were therefore taken into account for the choice of the deletion constructs. The shortest construct (PGL34_1–56_-GFP) only comprised 3 residues in addition to the predicted transit peptide and was designed as a stromal control. The second construct, comprising the amino acids 1–133 (PGL34_1–133_-GFP), contained the H1 domain as well as the conserved central domain. The construct PGL34_1–170_-GFP additionally included the H2 motif. The fourth construct (PGL34_1–290_-GFP) comprised H1, H2 and H3 motifs, as well as the conserved domain. An additional N-terminal deletion construct (PGL34_1–56..134–308_-GFP), lacking residues 57–133, contained the H2 and H3 motifs but not H1 and the central domain.

As shown in Fig. 2B, PGL34_1–56_-GFP and PGL34_1–133_-GFP gave diffuse signals broadly overlapping with the autofluorescence of the chlorophyll. In several chloroplasts however, peak GFP and chlorophyll fluorescence did not overlap, suggesting that both fusion proteins localised in the stroma. In contrast, when PGL34_1–290_-GFP construct was expressed in protoplasts, the fusion protein localised to small punctate structures, similarly to the full length protein. A distinct pattern was observed with PGL34_1–170_-GFP and PGL34_1–56..134–308_-GFP constructs. Fewer and larger fluorescent spots were observed in chloroplasts, suggesting mistargeting or protein aggregation. The same observation was made with constructs comprising residues 1–215 or 1–255 of PGL34 (not shown).

To verify the integrity of each GFP-fusion protein, transformed protoplasts were analysed by Western blotting using an anti-GFP serum (Fig. 2C). With the exception of PGL34_1–56..134–308_-GFP which could not be detected due to low expression levels, all GFP-fusion proteins migrated at the expected mass. Mistargeting (or aggregation) of PGL34 deletion constructs imply that the corresponding fusion proteins do not colocalise with full-length PGL34. We therefore cotransformed protoplasts with PGL34-CFP and PGL34 deletion constructs fused to YFP (Fig. 2D). As revealed in merged fluorescent images, CFP and YFP signals did not overlap in protoplasts expressing PGL34 and PGL34_1–133_. In contrast, PGL34-CFP and PGL34_1–290_-YFP colocalised, indicating that the short C-terminal hydrophilic part of the protein is dispensable for targeting. The strong punctate signals observed in protoplasts expressing PGL34_1–170 _were not labelled with PGL34-CFP, confirming mistargeting and/or aggregation of the truncated protein.

### Transgenic plants expressing YFP fused at the C-terminus of PGL34

**Figure 3:**
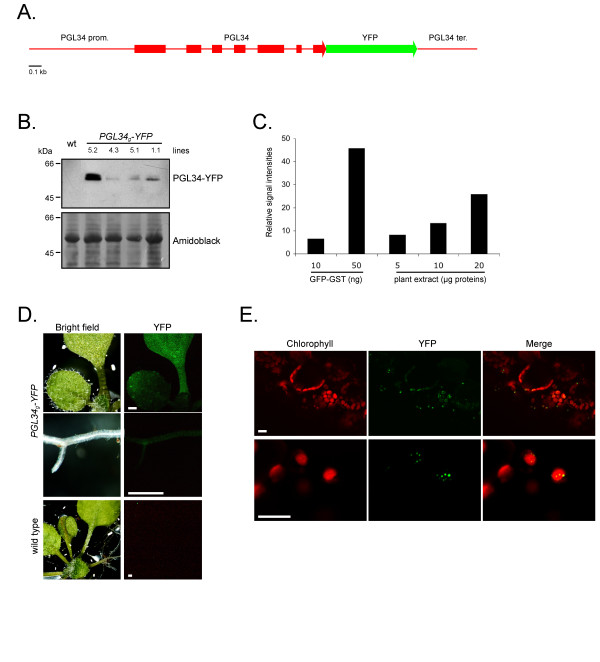
**Expression of PGL34_*g*_-YFP in planta**. A. Schematic representation of the construct used for plant transformation. The coding sequence of the yellow fluorescent protein (YFP) was inserted in the genomic sequence of *PGL34*, replacing the *PGL34 *stop codon. Boxes indicate coding regions. B. Western blot analysis of rosette leaves from homozygous T2 transgenic lines. PGL34-YFP was detected with anti-GFP antibodies. Proteins were visualized on the membrane by staining with amidoblack. Wt, wild type Arabidopsis plant protein extract. C. Yield estimation. Different amounts of total protein extracts from line 5.2 were subjected to SDS-PAGE in parallel with defined amounts of recombinant GST-GFP and chemiluminescent immunoblot signals were quantified. D. Detection of PGL34-YFP by fluorescence microscopy. YFP fluorescence was detected in leaves, cotyledons and roots by illuminating the seedlings with UV light. Scale bars: 0.5 mm. E. Subcellular localization of PGL34-YFP in leaves was determined using confocal laser scanning microscopy. Scale bars: 10 *μ*m.

The results obtained with PGL34-GFP deletion constructs suggested that most of the protein sequence is required for plastoglobule targeting. To address the potential of plastoglobule targeting for protein purification, Arabidopsis plants were transformed with the coding sequence of *YFP *replacing the stop codon in the genomic sequence of *PGL34 (PGL34*_*g*_-*YFP*, Fig. 3A). The chimeric coding sequence was placed under the control of *PGL34 *promoter and terminator in order to minimise the risk of gene silencing. Eleven primary transformants (Tl) were isolated and homozygous lines containing a single insertion locus were selected by segregation analysis (not shown). Expression of *PGL34*_*g*_-*YFP *in these lines was analyzed using both Western blotting and fluorescence microscopy. A wide range of transgene expression levels was observed (Fig. 3B), possibly reflecting positional effects of the T-DNA insertions [17]. A double band was detected in the transgenic plant extracts, the upper band possibly reflecting partial cleavage of the chloroplast targeting sequence.

The T2 line 5.2, accumulating the highest level of recombinant protein, was chosen for subsequent analysis. The concentration of recombinant protein in crude extracts was quantified by comparing immunoblot signals with serial dilutions of purified GFP produced in Escherichia coli (Fig. 3C). PGL34-YFP was calculated to account for approximately 0.2% (w/w) of total leaf proteins.

Strongest YFP fluorescence was detected in leaves (Fig. 3D) and roots showed comparably lower YFP signals, consistent with the expression pattern of *PGL34 *determined by microarray analysis [18]. A detailed picture of PGL34-YFP signals was obtained by confocal microscopy analysis of transgenic leaves (Fig. 3E). Punctate signals were observed in epidermal as well as in mesophyll cells. YFP patterns were similar to those observed in protoplasts transiently expressing *PGL34-YFP *(Fig. 3B), indicating targeting of the YFP cargo to plastoglobules *in planta*.

### Phenotype of plants accumulating PGL34-YFP

**Figure 4:**
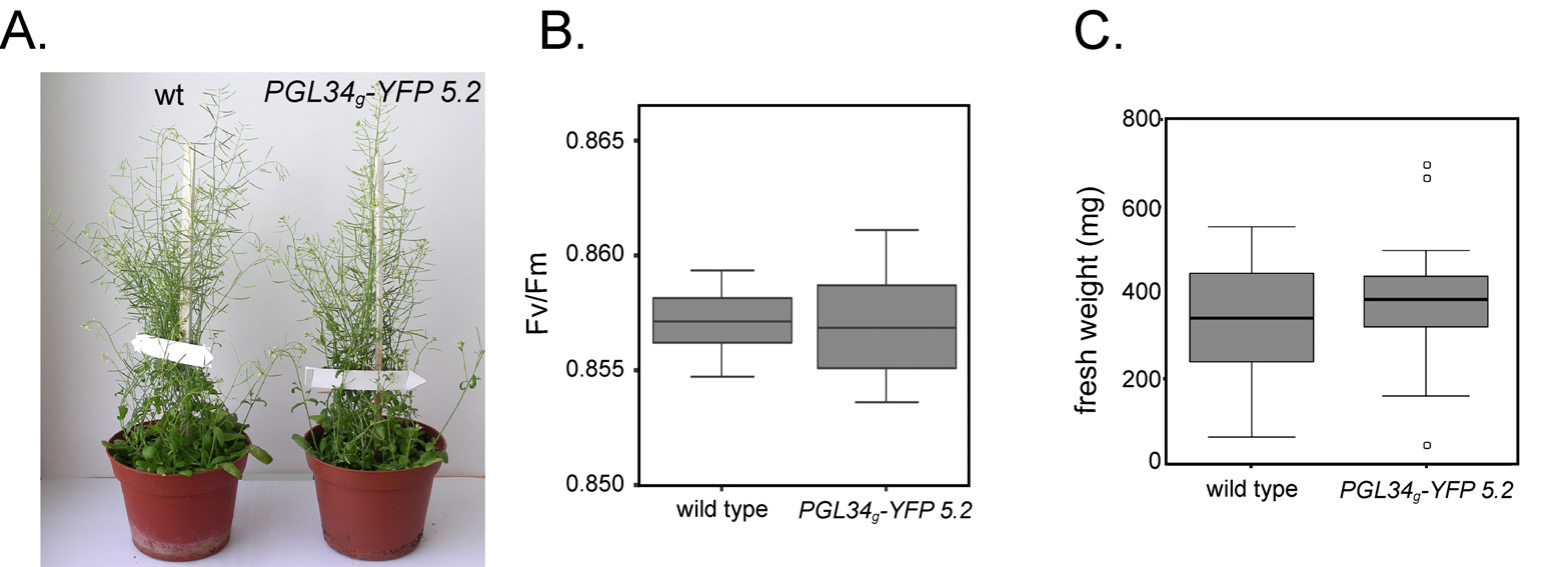
**Phenotype of plants expressing PGL34_*g*_-YFP**. A. Photographs of wild type (wt) or transgenic (line 5.2) plants grown for 8 weeks in 16/8 hours light/dark conditions. B. Maximum photosystem II quantum efficiencies (Fv/Fm) measured from rosette leaves. T-test, t = 0.17, p = 0.67, n = 14. C. Fresh weight measured 5 weeks after germination. T-test, t = -1.22, p = 0.23. At least 19 plants were measured. Normal distribution of the data (in B. and C.) was verified using the Kolmogorov-Smirnov test (p > 0.1).

Transgenic plants accumulating PGL34-YFP were indistinguishable from the wild-type when grown in short or long-day conditions (Fig. 4A). The germination rate of transgenic seeds was equal to wild type (Pearson Chi-Square test, n = 358, p = 0.33). No difference in fresh weight was observed between wild type and transgenic plants (Fig. 4C), indicating that growth was not altered in plants overexpressing *PGL34*_*g*_-*YFP*.

In sub-optimal photosynthetic conditions, the photosystem II (PSII) is subject to photoinhibition, characterised by a decrease of maximum PS II quantum efficiency (Fv/Fm, [19]). Fluorometry revealed similar Fv/Fm fluorescence values in transgenic vs. wild type leaves (Fig. 4B), ruling out major inhibitory effects of plastoglobule targeting on photosynthesis.

### Recombinant protein purification by flotation centrifugation

**Figure 5:**
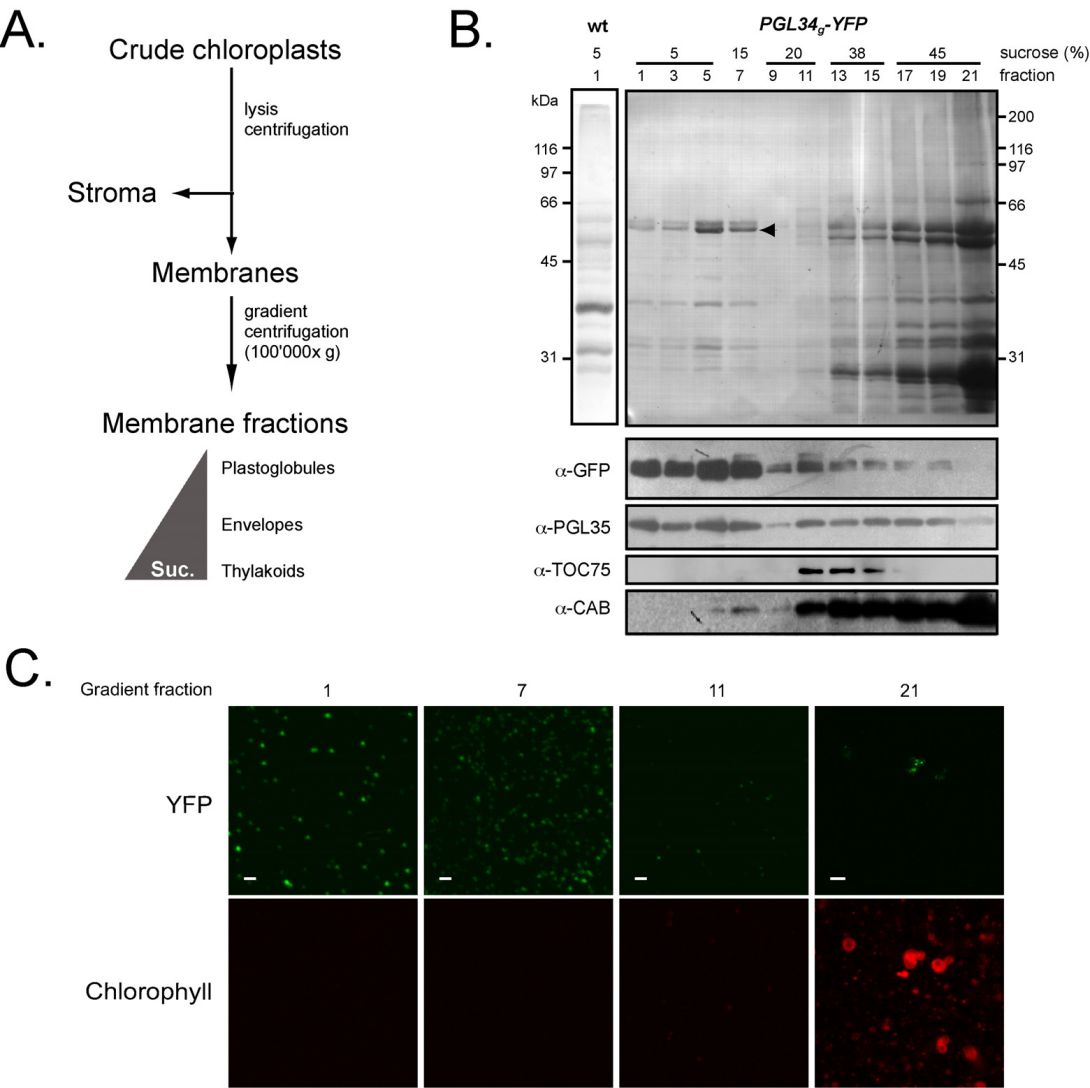
**Purification of PGL34-YFP by flotation centrifugation**. A. Diagram illustrating the purification procedure. Suc., sucrose concentration. B. Western blot analysis of fractions from the density gradient. After ultracentrifugation, 0.5 ml fractions were collected starting from the top of the gradient. Proteins contained in 400 *μ*l of fractions 1–7, 200 *μ*l of fractions 9–15, 100 *μ*l of fractions 17–19 or 50 *μ*l of fraction 21 were separated by SDS-PAGE, transferred to a nitrocellulose membrane and stained with amidoblack (upper panel). Silver stained plastoglobule proteins prepared from wild type plants (wt) are shown. Antibodies against GFP, PGL35, TOC75 and CAB were used for immuno blotting, as indicated (lower panels). C. YFP and chlorophyll fluorescence in fractions 1, 7, 11 and 21 was monitored by confocal microscopy. Scale bars: 1 *μ*m (fractions 1–11) or 5 *μ*m (fraction 21).

Plastoglobules were isolated from chloroplast membranes prepared from transgenic leaves using sucrose density gradient flotation centrifugation (see Fig. 5A). Fractions collected from the density gradient were analyzed by SDS-PAGE followed by Western blotting (Fig. 5B). The blots were probed with anti-PGL35 (At4g04020), anti-TOC75 (At3g46740) and anti-CAB (chlorophyll a/b binding protein) antibodies as plastoglobule, envelope and thylakoid markers, respectively. The results demonstrated the separation of plastoglobules (fractions 1–7) from envelope (in fractions 11–15) and thylakoid membranes (predominantly in fractions 11–21). PGL34-YFP, detected by anti-GFP antibodies, co-distributed with PGL35. Moreover, globular fluorescent structures were highly abundant in low-density fractions (Fig. 5C), demonstrating targeting of the recombinant protein to plastoglobules.

SDS-PAGE analysis of the low-density fractions revealed a prominent band with an apparent mass of 55 kDa consistent with the predicted mass of PGL34-YFP (28.3, processed PGL34 + 26.9, YFP). This band was recognized by anti-GFP antibodies (Fig 5B). Similar to immunodetection on total leaf extracts (Fig. 3a), an additional faint band, migrating at 57 kDa, was also recognized by the antibody. Analysis by tandem mass spectrometry confirmed that both bands contained PGL34-YFP (not shown). The upper band may reflect partial cleavage of the chloroplast targeting sequence or post translational modifications of PGL34-YFP. However, mass spectrometric analysis of tryptic peptides derived from the two protein bands yielded no additional evidence with regard to the two possibilities.

To estimate the enrichment factor resulting from the gradient flotation, the amount of recombinant protein in the 5% sucrose step (fraction nb. 6 of the gradient) was determined by comparing immunoblot signals with a dilution series of purified GFP (not shown). PGL34-YFP accounted for about 50% of the protein content. Based on this and the PGL34-YFP concentration in leaves (0.2% w/w), the gradient flotation step had lead to an approximately 250 fold enrichment of the recombinant protein.

### PGL34 homologues in cultivated plant species

**Figure 6:**
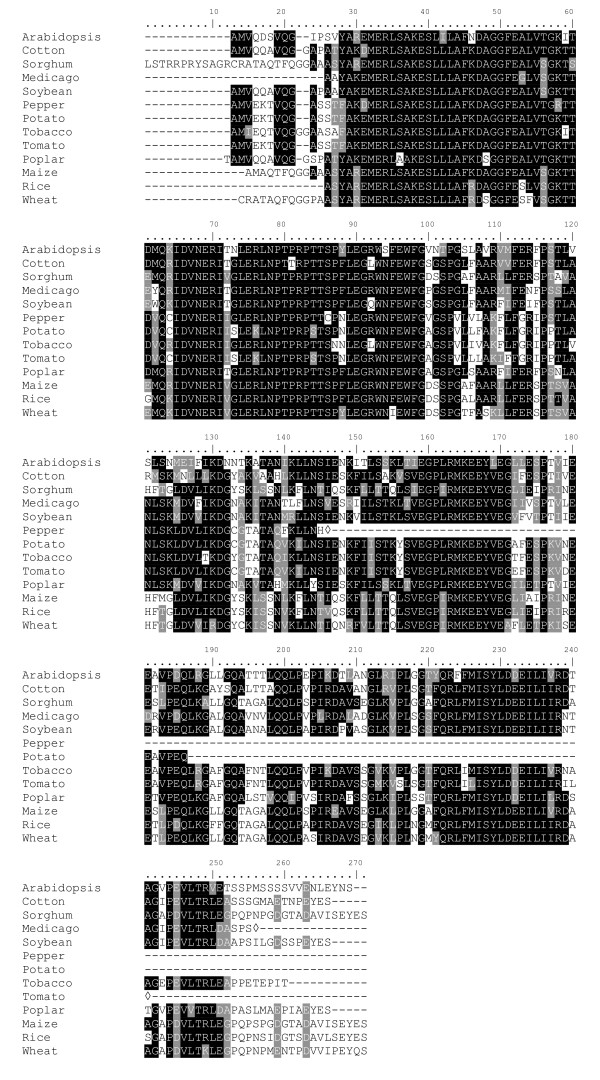
**Presence of PGL34 homologues in crop species**. Sequence alignment of proteins similar to PGL34. Amino acid sequences were aligned using ClustalW. Transit peptides were predicted using the TargetP software and removed from the protein sequences. The predicted transit peptide length of PGL34 was 53 amino acids. The alignment was formatted with the BOXSHADE 3.21 software. Residues identical in at least 6 sequences are shaded. Identical residues are in black and conserved substitutions are in grey. Diamonds indicate truncated sequences. EST contigs were retrieved from TIGR Plant Transcript Assemblies (TA, ) and Tentative Consensus (TC, ) databases. Cotton, TC61795; Sorghum, TC103242; Medicago, TC108241; Soybean, TC228644; Pepper, TC4347; Potato, TC120970; Tobacco, TA7467_4097; Tomato, TC164782; Poplar, TC38124; Maize, TC299788; Rice, AAO72625 (GenPept); Wheat, TC239733.

To determine whether proteins similar to PGL34 are present in cultivated plant species, sequence homology searches were performed. As shown in Fig. 6, protein sequences with significant BLAST hits were found in monocots and in a broad range of dicot taxa. Pairwise comparisons performed with the MatGAT software [20] revealed that the protein sequences shown in Fig. 6 were at least 40% similar. Arabidopsis PGL34 shared for example 79% similarity and 63% identity with the protein sequence deduced from a tobacco EST assembly. These data indicate that proteins from the PAP/fibrillin family are highly conserved in the plant kingdom.

## Discussion

### Sequence requirement for protein targeting to plastoglobules

The presence of proteins associated with plastoglobules implies that mechanisms ensuring protein assembly in plastoglobules must exist in plastids. However, nothing is known regarding these mechanisms and plastoglobulins do not share conserved sequence motifs with other plant or animal lipid body proteins. Several types of proteins associated with lipid bodies have been described in prokaryotic and eukaryotic cells (reviewed in ref. [21]). They have highly diverse physicochemical properties and topologies, reflecting various modes of association with the lipidic structures. In desiccation tolerant seeds, oil bodies are coated with oleosins [22]. A 72-residue central hydrophobic domain in the proteins, often referred to as "proline knot motif" is essential for their association with oil bodies [23-25]. In mammalian cells, under certain conditions, caveolins accumulate at the surface of cytoplasmic lipid droplets. Using deletion constructs, Ostermeyer et al. [26] demonstrated requirement of a hydrophobic domain for lipid droplet targeting of caveolin-1. Hydrophobic domains were also shown to play important roles in targeting and anchoring perilipin to lipid droplets in adipocytes [27]. If several lipid body proteins are characterised by hydrophobic domains, others lack large apolar regions. Adipophilin (also termed Adipocyte Differentiation-related Protein, ADRP), which localises to the periphery of cytosolic lipid bodies in mammalian cells, has no obvious lipid-binding motif (hydrophobic domains or amphiphatic *α*-helices; [21]) and discontinuous stretches of the protein are necessary for targeting to lipid bodies [28].

Although plastoglobulins associate with lipid bodies, their overall amino acid composition is not hydrophobic (Grand average of hydropathicity (GRAVY) index = -0.173 for PGL34) and it is apparent from hydropathy plots that the proteins, in contrast to oleosins, lack a strongly hydrophobic domain (see Fig. 3A). Therefore, association of PGLs with plastoglobules may rather rely on interactions with surface lipids, as proposed by Kim et al. [29], similarly to ADRP [21]. Based on our observation that the almost complete sequence of PGL34 is necessary to maintain proper targeting (Fig. 2), we propose that a correct folding of PAP/fibrillins rather than a sequence determinant is requested for assembly in plastoglobules.

### Similarities between plastoglobules and seed oil-bodies

A successful technology for production and recovery of recombinant proteins expressed in seeds from oilseed plants has recently been developed at SemBioSys [30,31]. In the so-called Stratosome™ system, proteins of interest fused to an oleosin moiety are targeted to oilbodies and recovered by subsequent centrifugation steps [32]. Biologically active human insulin has notably been produced from Arabidopsis seeds using this system [33].

Plastoglobules and seed oilbodies share similarities. They are of low density due to their high lipid content and only contain a few different proteins [14,15,34] – both properties being advantageous for purification by density partitioning. Whereas oleosin-coated lipid bodies accumulate predominantly in the cell cytoplasm of oleaginous seeds, in tapetum cells and in pollen grains [22], plastoglobules are ubiquitously found in all types of plastids [35], notably in leaf chloroplasts. In leaf crops such as tobacco producing high biomass, plastoglobule targeting would allow downstream processing of leaf material similar to the oilbody-oleosin system in seeds.

### Future perspectives

In this proof-of-concept study, a strong enrichment in PGL34-YFP was achieved. We expect that the yield of plastoglobulin-fusion proteins will be readily improved in follow-up studies using tissues accumulating plastoglobules or related fibrills such as fruits or senescing leaves [36-38] and/or using plastid genetic engineering [7]. The general usefulness of the plastoglobule-plastoglobulin system is predicted since plastoglobulins as well as plastoglobules are highly conserved throughout the plant kingdom and many homologues have been identified in genome and EST sequencing projects (Fig. 6). In particular leafy crop plants such as tobacco may be ideal to exploit the potential of the plastoglobule targeting system in molecular farming approaches.

## Conclusion

The data presented in this paper demonstrate the potential of protein accumulation in plastoglobules. Sequestration of recombinant proteins in the particles may limit deleterious effects on photosynthetic light and dark reactions. Plastoglobule accumulation combines the advantages offered by a chloroplast localization and, as in seed oil-bodies, of a simple enrichment step needed prior to standard chromatographic purification.

## Methods

### DNA vectors for plant transformation

The complete coding sequence of *PGL34 *(At3g58010), excluding the stop codon, was amplified by PCR from a cDNA clones (U15686) obtained from the Arabidopsis Biological Resource Center (ABRC, [39]). Forward (5'-cat gcc ATG GCA TTG ATC CAA CAT GG-3') and reverse (5'-cat gcc atg gcA CTG TTG TAT TCA AGA TTC TCT ACA AC-3') primers included *Nco*I sites. The PCR product was ligated in the *Nco*I site of either pCL60 [40] or pCL62 [14], resulting in C-terminal GFP or CFP fusions respectively, under the control of the CaMV 35S promoter and the nos terminator.

Partial *PGL34 *sequences were amplified from pCL60-PGL34 using a forward primer containing a *Xba*I site (5'-gct cta gaA TGG CAT TGA TCC AAC ATG G-3') and the following reverse primers, including *Nco*I sites: 5'-cat gcc atg gcA ACC ATA GCT CTG CAT ATC ATT C-3' (PGL34_1–56_), 5'-cat gcc atg gcA CTC CAT CTA CCT TCA AGA TAA GG-3' (PGL34_1–133_), 5'-cat gcc atg gcA TCT TTA ATG AAT ATT TCC A-3' (PGL34_1–170_), 5'-cat gcc atg gcT TCC ACC CTC GTT AGA AC-3' (PGL34_1–290_). The corresponding PCR products were ligated into the *Xba*I and *Nco*I sites of pCL60 or pCL61 [14], yielding GFP and YFP fusions, respectively. To obtain the deletion construct pCL60-PGL34_1–56..134–308_, a C-terminal fragment of *PGL34 *was amplified from pCL60-PGL34 using forward (5'-cat gcc atg gcc TTT GAG TGG TTT GGA GTC AAC-3') and reverse (5'-cat gcc atg gcA CTG TTG TAT TCA AGA TTC TCT ACA AC-3') primers including *Nco*I sites and ligated in the *Nco*I site of pCL60-PGL34_1–56_. For stable plant transformation, a genomic DNA fragment, including PGL34 coding sequence as well as a 0.85 kb 5' upstream region was amplified by PCR using forward 5'-CAT GCC ATG GAG ATC TTC GGT GAG GAA CAA GAG TT-3' and reverse 5'-CAT GCC ATG GCA CTG TTG TAT TCA AGA TTC TCT ACA AC-3' primers and inserted in the *Nco*I site of the pCL61 vector, in frame with the coding sequence of YFP. PGL34 3' downstream region (0.47 kb, including the terminator) was amplified by PCR from genomic DNA using forward 5'-CCG CGG CCG CAA ACA GGT TCT CTT GTT ACT CTG ATT C-3' and reverse 5'-GGG CGG CCG GAG ATC TCG GTC TCT CAA AGG ATG TG-3' primers. The fragment was cloned in the *Not*I site of pCL61. PGL34 and YFP sequences were excised from pCL61 by restriction digest with *Aat*II and *Kpn*I. Fragments were blunted and ligated in the *Sma*I site of pCAMBIA3300 binary vector (CAMBIA), yielding pCAMBIA3300:PGL34-YFP.

### Transient and stable A. thaliana transformation

Transient transformation of protoplasts was done using the polyethylene glycol method as described in ref. [41], but reducing cellulase and macerozyme (Serva) concentrations to 1% and 0.25% (w/v) respectively. Fluorescence in transformed protoplasts was monitored 48 h after transformation by confocal laser scanning microscopy. GFP was detected using the FITC (488 nm) laser line from a LEICA TCS 4D microscope (LEICA Microsystems). For double fluorescent experiments, CFP and YFP were detected sequentially with a LEICA SP2 AOBS microscope, using 458 and 514 nm laser lines and 460–510 nm and 520–588 nm detection windows, respectively. Chlorophyll autofluorescence was monitored using either 594 nm or TRITC (568 nm) excitation wavelengths.

Stable transformation of *A. thaliana *plants with pCAMBIA3300:PGL34-YFP was carried out using the floral dip method as described [42,43]. Transformants were selected on plates containing phosphinothricin.

### Protein extraction and immunoblot analysis

Proteins were isolated from Arabidopsis leaves according to ref. [44]. 25 *μg *proteins were concentrated by chloroform – methanol precipitation [45], separated by SDS-PAGE and blotted onto nitrocellulose membranes. Proteins were stained with amidoblack (= naphthol blue black) for protein detection or used for immunodetection. Blots were probed with anti-GFP (gift from Dr. E. Schäfer) and anti-CAB (kindly provided by Dr. K. Apel) sera, or with affinity-purified antibodies specific for TOC75 [46] and PGL35 [14]. For quantification, chemiluminescent immunoblot signals were analysed using a Bio-Rad ChemiDoc XRS system.

### Gradient flotation centrifugation of chloroplast membranes

Leaves from Arabidopsis plants grown on soil for 4 weeks were harvested and immersed in tap water in the dark at 4°C for 30 min. Leaf material was homogenized in HB buffer (450 mM sorbitol, 20 mM Tricine/KOH pH 8.4, 10 mM EDTA, 10 mM NaHCO_3_, 1 mM MnCl_2_) with a Waring blender and filtered through cheese cloth and miracloth. Chloroplasts were sedimented (2 min 700× g), washed with TrE (50 mM Tricine/HCl pH 7.5, 2 mM EDTA) and hypertonically lysed 10 min in TrE + 0.6 M sucrose supplemented with 0.5% (v/v) protease inhibitor cocktail (Sigma P9599). The lysate was frozen at -80°C, thawed on ice, diluted 3 times with TrE buffer and homogenised with a Potter homogeniser. Total membranes, corresponding to 10 mg of chlorophyll, were sedimented at 100'000× g and resuspended in 3 mL 45% sucrose in TrE buffer. Membranes were overlaid with a discontinuous sucrose gradient consisting of 2 ml 38% sucrose, 2 ml 20% sucrose, 1.4 ml 15% sucrose and 2.7 ml 5% sucrose in TrE buffer and centrifuged for 17 h at 100'000× g and 4°C (SW41Ti rotor, Beckman). 0.5 ml fractions were collected starting from the top of the gradient and used for confocal microscopy analysis or Western blotting (see above).

### Fluorometry

Maximum quantum efficiency of photosystem II (Fv/Fm) was measured using a Handy Plant Efficiency Analyser chlorophyll fluorometer (Hansatech Instruments, Norfolk, UK). Detached leaves from 4 week-old plants were dark-adapted 20 min prior to measurements.

## Authors' contributions

PV carried out most of the cloning work, transient and stable plant transformation, as well as the microscopic and biochemical analyses and helped to draft the manuscript. CB participated in the elaboration of the experimental design and did the sequence analysis. FK conceived the study, drafted the manuscript and was responsible for the research program. All authors read and approved the final manuscript.
